# Minimal important change or difference for the oxford hip and knee scores following joint replacement surgery

**DOI:** 10.1186/1745-6215-14-S1-O51

**Published:** 2013-11-29

**Authors:** Kristina Harris, Andrew Price, Jill Dawson, Helen Doll, David Murray, Andrew Carr, David Beard

**Affiliations:** 1 University of Oxford, Oxford, UK

## Objectives

i) to present a range of estimates of meaningful or minimal important changes/differences for the Oxford Hip Score (OHS) and the Oxford Knee Score (OKS) based on different approaches, and (ii) to identify the estimates that are most consistent and useful for application in specific contexts.

## Study design and setting

Secondary data analysis of the NHS PROMS dataset which included 137,109 patients listed for hip replacement surgery and 156,788 patients listed for knee replacement surgery.

## Results

Anchor based Minimal Important Difference (MID) was ~ 5 points for the OKS and the OHS and Minimal Important Change (MIC) was ~ 9 points for the OKS and ~ 11 points for the OHS. Distribution based methods showed that the Minimal Detectable Change (MDC_90_) for the OKS and OHS respectively were 4 and 5 points.

## Conclusion

This study produced a range of context-specific estimates of minimal important change/difference for the OKS/OHS. We would recommend, based on current evidence, that the OKS/OHS MIDs are used to indicate meaningful difference between patients (e.g. in clinical trials) and the OKS/OHS MICs to indicate meaningful change from baseline in a single group design. The MDC_90_ should be used to indicate change beyond measurement error on individual patients.

